# Evaluation of dose delivery accuracy of the lung with the respiratory technique of the Radixact Synchrony real-time tumor tracking system

**DOI:** 10.1016/j.tipsro.2025.100316

**Published:** 2025-05-24

**Authors:** Evren Ozan Göksel

**Affiliations:** Acibadem MAA University, Vocational School of Health Services, Radiotherapy Program, Istanbul, Turkey; Acibadem Altunizade Hospital, Department of Radiation Oncology, Istanbul, Turkey

**Keywords:** Radixact Synchrony, Lung SBRT, Motion management, Markerless target tracking

## Abstract

**Purpose:**

This study aims to evaluate the dosimetric accuracy of the Lung with Respiratory (LWR) technique in the Radixact Synchrony real-time tumor tracking system using data from actual patients undergoing lung SBRT.

**Material and methods:**

This retrospective study included 20 patients treated with the Synchrony LWR technique for primary lung cancer or lung metastases. Mid-ventilation breath-hold CT and 4DCT images were acquired for treatment planning and tumor motion evaluation. Gamma analysis was performed using the PTW 1500 detector. Three verification plans were generated for each patient: one for gamma analysis without motion (GAWoM), one for point dose measurement with motion (PDWM), and one for gamma analysis with motion (GAWM). Patient specific respiratory motion was simulated using a CIRS motion platform. The relationship between respiratory patterns, treatment plan parameters, and the dose delivery accuracy of the Synchrony system was evaluated using correlation analysis.

**Results:**

The PDWM results demonstrated a strong agreement between the planned and measured doses, with a maximum difference of less than 2%. The gamma passing rates of 3D dose measurements for all patients’ Synchrony plans were found to be within the clinically acceptable level. While GAWM results were slightly lower than GAWoM results, correlation analysis revealed a strong positive relationship between the two. A moderate negative correlation was observed between the modulation factor and GAWM, similar to GAWoM. Additionally, a strong negative correlation was observed between beam-on time and both GAWoM and GAWM. Furthermore, no significant correlation was found between GAWM and respiratory pattern-related variables, including superior-inferior and lateral motion amplitude or respiratory rate per minute.

**Conclusion:**

The results of the correlation analyses indicate that the dose delivery accuracy of the Synchrony LWR technique is comparable to that of static target irradiation, independent of the patient’s respiratory pattern or the planning parameters used.

## Highlights

• No significant correlation was observed between the measurement results and variables related to respiratory patterns.
• A strong positive correlation was observed between the results of gamma analysis with motion and without motion.
• The gamma passing rates for the Synchrony plans of all patients were within the clinically acceptable level.

## Introduction

Stereotactic body radiotherapy (SBRT) provides good local control in patients with primary or metastatic thoracic malignancies. [[1], [2], [3]]. In these treatments, motion management techniques are essential to reduce the uncertainty in delivering high fractional doses to the target [4,5]. Various methods are used for clinical motion management, including respiratory-gated radiation delivery, breath-holding techniques, abdominal compression to limit respiratory amplitude, and tumor tracking [6]. Studies have demonstrated that some of these techniques may not be well tolerated by patients, which could potentially affect clinical outcomes [[7], [8], [9], [10], [11]]. For instance, respiratory-gated radiation delivery can significantly extend treatment time, while breath-holding techniques may not be well tolerated by patients with pulmonary issues [12,13]. In such cases, as an alternative method, the internal target volume (ITV) is determined using 4D imaging modalities to ensure that the tumor receives the prescribed dose while accounting for respiratory-induced motion. However, using the ITV approach often results in a larger volume of normal tissue being exposed to high doses of radiation [14,15].

With the new technology, the “Synchrony Real-Time Tumor Tracking System” (Radixact®, the next-generation TomoTherapy® System; Accuray Incorporated, Sunnyvale, CA), the motion of the target structure can be tracked through the movement of the jaws and multi leaf collimator (MLC). This technology enables the reduction of additional ITV margins around the tumor, thereby minimizing the volume of normal tissue exposed to radiation [16]. It has been previously demonstrated that lung SBRT plans created using Radixact Synchrony and CyberKnife Synchrony, without applying the ITV approach, are dosimetrically comparable in terms of conformity index, homogeneity index, and OAR sparing [17].

The Radixact Synchrony system can be used with two different techniques to compensate for tumor motion caused by respiration. The first technique, “Lung with respiratory (LWR),” compensates for respiratory motion of solid tumors visible on kilovoltage (kV) radiographs in SBRT treatments for lung cancer patients. In this technique, the patient's respiratory motion is tracked using infrared light-emitting diodes (LEDs) placed on the patient's body. Simultaneously, it acquires radiographic images from different gantry angles and locks onto the tumor in these images. The Synchrony system combines these two sets of data to create a model that predicts the tumor's future location. The model is updated with each new kV radiographic image. Since tumor-tracking treatment with Synchrony is conducted during free breathing, the treatment duration is similar to that of conventional treatments using the ITV approach.

The second technique, “Fiducial with respiratory (FWR),” is applicable for treating other tumors located in organs like the lung or liver, which are affected by respiratory motion but are not visible on radiographs, provided fiducial markers are placed. In this technique, fiducial markers are tracked in radiographic images instead of the tumor, assuming that the target moves in synchronization with these fiducials. Simultaneously, respiratory motion data from the LEDs is utilized, similar to the LWR technique, to track the movement of the target.

In our clinical practice, the LWR technique is used as the motion management method for lung SBRT applications. This study aims to evaluate the dosimetric accuracy of the LWR technique of the Radixact Synchrony real-time tumor tracking system using data on tumor size, respiration-induced tumor motion, and treatment parameters from actual patients.

## Material and methods

### Patient setup and data acquisition

The treatment plans and respiratory pattern parameters of our first 20 patients treated for primary lung cancer or lung metastases using the Synchrony LWR technique between November 2023 and August 2024 were retrospectively used. The tumor was centrally located in 10 patients and peripherally located in 10 patients. Before deciding on treatment with Synchrony, a simulation plan was created for all patients, and a treatment simulation was conducted to assess whether the target's position, density, and motion characteristics were suitable for treatment using Synchrony LWR technique. During the treatment planning process, for patients deemed suitable for treatment with this technique, both four-dimensional computed tomography (4DCT) and mid-ventilation breath-hold computed tomography (MVBH-CT) images were acquired in the supine position (Siemens Go Up − Varian Respiratory Gating for Scanners, Varian, Palo Alto, CA, USA). The MVBH-CT image was obtained within a region of free breathing, and both contouring and treatment planning were performed using this CT image. This phase does not involve a breath-hold in deep inspiration, which would deviate the patient from their normal respiratory pattern. The 4DCT was used solely to assess the tumor’s respiratory motion and was not utilized for treatment planning. Since the MVBH-CT and 4DCT images were acquired consecutively, no additional registration of the images was necessary. The images were automatically imported into the planning system in DICOM-registered format. The 4DCT was quantitatively evaluated, and any discrepancies between the tumor position contoured on the MVBH-CT image and those contoured on the 4DCT image across all phases of free breathing were assessed. These procedures were performed to confirm that the respiratory phase during which the MVBH-CT image was acquired corresponds to a phase of free breathing. In the patients included in the study, no discrepancies were observed between the tumor positions contoured on the MVBH-CT and those on the 4DCT images.

### Treatment planning

Treatment planning was performed using the Accuray Precision treatment planning system (TPS) (Accuray, Sunnyvale, CA, USA) with the VoloUltra optimization algorithm. Dose calculations were conducted using the Collapse Cone Convolution Superposition (CCCS) algorithm. The field size was set to 2.5 cm, while the modulation factor (MF) was automatically determined by the TPS (range 1–2.5). The pitch factor was set to ensure the optimum gantry period required for the Synchrony technique. The prescribed doses and plan parameters were shown in Table 1 for each patient. Since different fractionation schemes were used in our study, the ratio of treatment time to fraction dose (TTDF) was evaluated alongside the beam-on time. TTDF enables normalization of treatment durations according to dose and allows for a dose-adjusted evaluation. Additionally, the TTDF parameter is recognized as a simple indicator that provides insight into plan complexity [18]. In the evaluation of target coverage, gradient index, and dose to organs at risk in SBRT treatment planning, the UKSABR 2022 guideline was used as the basis [19]. In patients with ultra-central tumors or those undergoing second-course irradiation, a more protective approach was adopted, deviating from the UK SABR 2022 guidelines, with dose schedules involving 10 fractions. Nevertheless, all treatment plans were designed in accordance with the SBRT planning technique, ensuring a heterogeneous dose distribution within the target and a rapid dose fall-off beyond the target boundary.

**Table 1:** Treatment planning parameters and patient-specific plan verification results for each patient.

Patient Number	PTV (cc)	Fraction Dose (Gy)	Fraction number	MF	Pitch	Mean- LOT (ms)	Beam-on time (s)	TTDF s/cGy	GAWoM %	GAWM %	PDWM %
1	2.7	16.0	3	1.1	0.06	418.5	595.1	0.4	90.9	90.0	1.9
2	14.7	7.0	8	1.1	0.08	293.0	601.1	0.9	91.2	90.1	0.9
3	80.3	5.0	10	2.1	0.08	120.1	585.3	1.2	92.0	91.7	−1.4
4	16.1	11.0	5	1.0	0.09	1125.0	569.4	0.5	99.0	98.5	0.2
5	10.2	11.0	5	2.5	0.07	312.2	610.2	0.6	92.0	91.9	1.9
6	12.8	5.0	8	1.1	0.09	230.2	344.3	0.7	93.8	93.4	0.5
7	4.3	11.0	5	1.1	0.07	494.3	798.8	0.7	93.0	92.3	0.2
8	5.7	10.0	5	1.1	0.07	261.6	417.6	0.4	92.5	92.3	0.7
9	4.3	10.0	5	1.1	0.09	248.1	288.9	0.3	94.0	93.4	−0.6
10	16.2	10.0	5	1.1	0.07	212.0	400.2	0.4	100.0	100.0	0.7
11	3.3	10.0	5	1.2	0.05	281.9	509.2	0.5	93.1	92.2	1.1
12	85.0	7.0	8	1.4	0.07	173.2	577.5	0.8	92.0	91.0	1.7
13	28.9	5.0	10	1.2	0.10	203.7	447.6	0.9	100.0	98.3	−0.8
14	37.8	5.0	10	1.2	0.06	205.1	538.4	1.1	100.0	97.8	0.6
15	35.3	5.0	8	1.1	0.13	369.7	481.2	0.9	98.7	92.3	1.1
16	19.7	7.5	8	1.1	0.07	224.2	340.2	0.5	100.0	99.1	1.2
17	55.5	7.5	8	1.6	0.10	211.5	481.0	0.6	92.2	91.5	1.7
18	3.1	10.0	5	1.1	0.10	225.9	266.6	0.3	99.8	93.4	−0.8
19	35.3	4.5	10	1.5	0.07	154.9	485.2	1.1	93.8	92.6	1.1
20	13.1	8.0	5	1.2	0.13	385.1	311.6	0.4	95.7	95.0	1.0

### Patient specific plan verifications

In our clinical practice, patient-specific treatment plan verification using 3Dimensional (3D) gamma analysis is performed for all patients undergoing treatment with helical tomotherapy technique, utilizing the PTW Octavius phantom and the PTW 1500 detector (PTW-Freiburg, Freiburg Germany).

The PTW 1500 detector system operates with ion chambers, which differentiates it from the diode detector systems employed in other studies. The PTW 1500 detector array consists of 1405 vented ionization chambers, each with an entrance area of 4.4 × 4.4 mm^2^ and a height of 3 mm, resulting in an ionization volume of 0.058 cm^3^. The chambers are arranged in a checkerboard pattern, with every second row shifted by 5 mm in the row direction relative to its adjacent rows. This configuration results in a row-to-row distance of 5 mm and a nearest-neighbor spacing of 7.1 mm along the diagonal. The PTW array provides a spatial sampling frequency of 0.1 mm^−1^ in both the row and column directions, which corresponds to a detector spacing of 10 mm. Along the diagonal, the sampling frequency increases to 0.14 mm^−1^ due to geometric projection. By combining two measurements acquired with a 5 mm lateral or longitudinal shift of the array, the effective sampling frequency in the row and column directions can be improved to 0.2 mm^−1^.Stellijies et al. demonstrated that when the detector PTW 1500 is used for intensity modulated radiotherapy plan verification, the results exhibit a high level of agreement with EBT3 film measurements, similar to those obtained with the detector PTW 1000SRS [20]. To improve resolution, the merge feature was applied in all measurements conducted with the PTW 1500 detector [20,21].

Thin-slice CT images (2 mm slice thickness) of the Octavius phantom were acquired with the PTW 1500 detector in place and imported into the Accuray Precision TPS. The clinical plan was copied onto this phantom CT to generate a patient-specific verification plan. The 3D dose distribution calculated for the phantom was exported in DICOM format and imported into Verisoft software (Version 8, PTW-Freiburg). The measured dose was compared with the calculated distribution using the 3D gamma analysis tool in Verisoft. Although TG-306 recommends using relaxed gamma criteria (3 %/3 mm) for motion-tracking systems, we applied the stricter TG-218 criteria (3 % global DD, 2 mm DTA, 10 % threshold) for both motion and non-motion analyses [22,23].

Each treatment plan was first subjected to standard three-dimensional (3D) gamma analysis without motion (GAWoM) as part of routine clinical quality assurance. Thus, the analysis of static verifications enables us to determine the planned-irradiated dose distribution mismatch caused by the use of high MLC modulation in the treatment plan. If the gamma pass rate for this evaluation is found to be above 90 %, patient-specific QA plans are created using a moving phantom designed to simulate respiratory motion, as recommended by AAPM TG-101 [24]. If the evaluation result fails, the process returns to the planning stage, where efforts are made to reduce the complexity of the plan.

To actively utilize the Synchrony LWR technique during measurements, it is essential to position a high-density material near the center of the phantom, which can be defined as a tracking volume and detected in the kV radiographs by the system. Therefore, for patient-specific verifications involving respiratory motion, the Cheese phantom (Tomophant, Accuray) was used instead of the Octavius phantom due to the inability to position a high-density material near its center. During the CT acquisitions and measurements, a high-density plug (1.5 g/cm^3^) was placed in the hole closest to the center of the Cheese phantom.

**Fig. 1:**
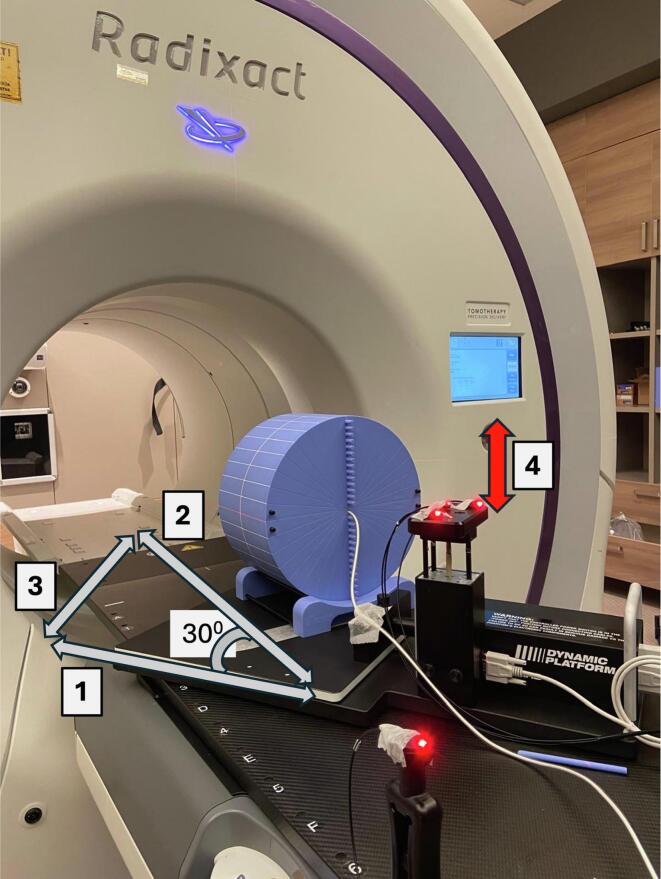
The setup for PDWM measurements simulating respiratory motion using the Cheese phantom, the A1SL ion chamber, and the CIRS dynamic motion platform. (1) Motion direction of the platform, (2) Superior-inferior motion direction of the Cheese phantom, (3) Lateral motion direction of the Cheese phantom, (4) Surrogate section (LEDs) motion direction.

**Fig. 2:**
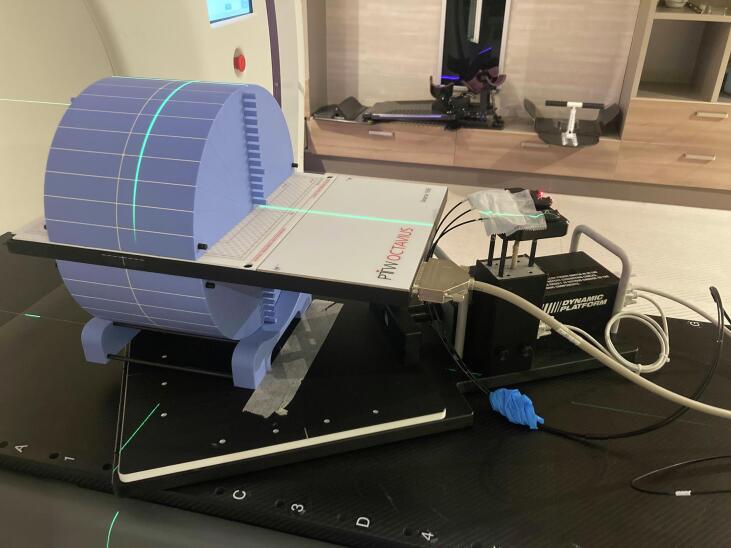
The setup for GAWM measurements simulating respiratory motion, utilizing the Cheese phantom, the PTW 1500 detector, and the CIRS dynamic motion platform.

While 3D gamma analysis offers a broader assessment of dose coverage across the entire treatment volume, it allows for a relative evaluation of the agreement between the delivered and planned dose distributions. However, to ensure that there are no significant deviations in the absolute dose delivered to critical points, such as the target, it is recommended to supplement it with point dose measurements [23,25,26]. Therefore, both 3D gamma analysis and point dose verification were performed in our study. To facilitate these measurements, the Cheese phantom was placed on the CIRS dynamic motion platform (Model 008PL, Computerized Imaging Reference Systems, Inc.). Thin-slice CT images (2 mm) were acquired in two separate setups: one with the A1SL ionization chamber (Standard Imaging) inserted into the phantom, and another with the PTW 1500 detector positioned between two sections of the phantom. Both CT images were imported into the TPS as phantom data. The active part of the A1SL ion chamber was contoured on the CT images acquired with the ion chamber. The plug was defined as the Tumor Tracking Volume (TTV) to enable Synchrony tracking [27]. Two verification plans were generated per patient: one for absolute point dose verification (Fig. 1) and one for dose distribution verification (Fig. 2). After the verification plans were created, the mean dose value calculated for the contoured ion chamber volume was used as the planned point dose. Before operating the CIRS motion platform, a megavoltage CT image of the measurement setup was acquired, and setup errors were corrected accordingly. While the CIRS system simulated patient-specific respiratory motion, the Synchrony LWR technique actively compensated for this motion. The percentage difference between the measured absorbed dose (point dose measurement with motion, PDWM) and the planned dose was calculated. The dose data obtained from the patient-specific verification plan, generated using CT images of the measurement setup with the Cheese phantom and PTW 1500 detector, were compared with the detector-measured dose using gamma analysis (gamma analysis with motion, GAWM).

The Synchrony LWR technique allows for kV radiographic imaging from up to six different angles per gantry rotation. When creating verification plans, four distinct kV radiographic angles were specified for each plan. The reason for optimizing the number of kV radiographs taken during each gantry rotation was to minimize the dose contribution from these imaging procedures while still achieving a robust respiratory model. In this study, the impact of dose contribution from kV radiographic imaging was not evaluated.

### The use of a respiratory motion-simulating platform to simulate patient-specific respiratory patterns

For each patient, the respiratory rate and amplitude values obtained from the infrared-reflective surrogate used during the 4DCT scan were directly entered into the control software of the CIRS Dynamic Platform. These values were then used to reproduce the surrogate motion in the CIRS simulation system, where the LEDs were positioned on the moving section. To measure tumor motion in the superior-inferior direction, the center of mass shift values in the longitudinal direction for the GTV structures delineated in the 10 phases were evaluated. This evaluation was performed using the DICOM Statistics tool in the contouring section of the Eclipse treatment planning system (version 16, Varian, Palo Alto, CA, USA). The required motion of the CIRS platform in the direction of movement was calculated to determine the patient-specific superior-inferior motion amplitude of the tumor. Due to the CIRS system's platform, where the phantom is placed, being limited to movement in only one direction (1D), lateral motion could not be customized for each patient. However, since the CIRS platform was yawed by 30 degrees around the IEC Z-axis (rotated relative to the IEC Y-axis), a lateral (IEC X) motion proportional to the superior-inferior (IEC Y) movement was also induced in the Cheese phantom (Fig. 1). For each patient, patient-specific respiratory information was entered into the CIRS system's software during point dose and GAWM measurements (Table 2). In this study, both the surrogate section and the platform in the CIRS system exhibited one-dimensional sinusoidal motion. During the verification measurements, while the CIRS system was simulating patient-specific respiratory motion, the Synchrony tumor tracking system was actively tracking this motion. The imaging parameters for kV radiographs were set to predefined values for a medium-sized thorax image: 1.0 mAs and 120 kV.

**Table 2:** Information regarding the patients' respiratory patterns.

Patient Number	Surrogate section (LEDs) Motion Amplitude (mm)	Respiratory Rate per Minute	Sup-inf motion amplitude of tumor in real patient (mm)*	Motion amplitude of Motion platform (mm)**	Lateral motion amplitude of the phantom (mm)***
1	6.0	20	6.0	7.0	3.5
2	9.0	18	10.0	11.6	5.8
3	14.0	15	15.0	17.4	8.7
4	5.0	25	10.0	11.6	5.8
5	7.0	16	5.0	5.8	2.9
6	10.0	20	5.0	5.8	2.9
7	10.0	19	8.0	9.3	4.6
8	10.0	15	16.0	18.6	9.3
9	8.0	12	5.0	5.8	2.9
10	7.0	18	3.0	3.5	1.7
11	22.0	10	7.0	8.1	4.0
12	16.0	14	10.0	11.6	5.8
13	6.0	19	5.0	5.8	2.9
14	10.0	20	6.0	7.0	3.5
15	8.0	21	15.0	17.4	8.7
16	9.0	12	7.0	8.1	4.0
17	9.0	13	12.0	13.9	6.9
18	14.0	10	18.0	20.9	10.4
19	8.0	23	15.0	17.4	8.7
20	7.0	14	10.0	11.6	5.8

*The patient-specific superior-inferior motion amplitude of the tumor measured using 4DCT images.

**The motion amplitude calculated for the movement of the CIRS motion platform.

***The calculated lateral motion amplitude of the phantom resulting from the placement of the CIRS motion platform at a 30-degree angle to the table’s longitudinal axis.

### Statistical analysis

According to the Shapiro-Wilk test, the variables planning target volume (PTV), LED motion amplitude, modulation factor, GAWM, and GAWoM did not exhibit a normal distribution. Therefore, the Spearman non-parametric test was used for the correlation analysis.

## Results

Table 1 presents the PTV volumes, patient-specific prescribed doses, treatment planning parameters and plan verification results for each patient. The median PTV volume was 15.40 cc (range: 2.68 cc to 85.00 cc). The median pitch value used in the treatment plans was 0.08 (range: 0.05 to 0.13), and the median MF was 1.1 (range: 1.0 to 2.5). The values provided in the table for PDWM represent the percentage differences between the planned and measured doses, while the values for gamma analysis represent the percentage gamma passing rates between the planned and measured dose distributions. The absolute difference between the planned and measured PDWM values had a median of 0.9 %, with a maximum of 1.9 % and a minimum of 0.2 %.

The median values for the gamma analysis results, both with and without accounting for respiratory motion, were found to be similar. The values were 95.1 (90.9, 100.0) for GAWoM and 93.8 (90.0, 100.0) for GAWM.

The leaf open times (LOT) values in the treatment plans were analyzed, revealing an average of 364.3 ms ± 210.3 for max-LOT and an average of 307.5 ms ± 213.6 for mean-LOT. Additionally, the average gantry period was found to be 17.0 s ± 5.2 (range: 11.8–28.8). For the treatment plans included in our study, the mean TTDF value was 0.7 s/cGy ± 0.3 (range: 0.3–1.2).

The parameters used to simulate patient-specific respiratory motion in plan verification measurements for each patient are presented in Table 2. These parameters include LED motion amplitude, respiratory frequency, and the values representing the tumor's superior-inferior displacement. All amplitude values reported in this study refer to peak-to-peak amplitudes. The median respiratory rate was found to be 17 breaths per minute (range: 10 to 25), while the median superior-inferior and lateral motion amplitudes were determined to be 8 mm (range: 3 mm to 18 mm) and 4.6 mm (range:1.7 mm to 10.4 mm), respectively.

Table 3 presents the correlations and statistical significance among the GAWM, PDWM, and GAWoM results and various parameters, including the prescribed dose, modulation factor (MF), pitch value, and the extent of target motion in the superior-inferior and lateral directions.

**Table 3:** Correlation coefficients (r) and significance levels (p) between gamma analysis results and point dose measurements with motion and other variables. Bold values indicate statistically significant moderate or strong correlations.

Other Variables	GAWM vs.		PDWM vs.		GAWoM	
	Correlation Coefficients (r)	Significance levels (p)	Correlation Coefficients (r)	Significance levels (p)	Correlation Coefficients (r)	Significance levels (p)
LED motion Amp.	−0.201	0.395	0.018	0.939	NA	NA
Respiratory Rate per Min	0.133	0.575	−0.312	0.181	NA	NA
Sup-inf motion amplitude	−0.306	0.189	0.160	0.501	NA	NA
Lateral motion amplitude	−0.300	0.199	0.157	0.508	NA	NA
PTV Volume	0.047	0.845	0.233	0.322	0.125	0.599
Dose	−0.078	0.743	−0.054	0.822	−0.201	0.395
MF	−0.446*	0.049	0.693**	0.001	−0.445*	0.049
Pitch	0.195	0.410	−0.149	0.532	0.241	0.306
Beam ontime	−0.623**	0.003	0.263	0.263	−0.600**	0.005
TTDF	−0.216	0.359	0.133	0.576	−0.123	0.605
Gantry period	−0.223	0.346	−0.079	0.740	−0.241	0.306
Max-LOT	−0.210	0.375	−0.088	0.714	−0.230	0.330
Mean-LOT	−0.075	0.752	−0.217	0.359	−0.156	0.512
Point dose difference	−0.593**	0.006	1	NA	−0.501*	0.024
GAWM	1	NA	−0.593**	0.006	0.936**	0.000
GAWoM	0.936**	0.000	−0.501*	0.024	1	NA

**Correlation is significant at the 0.01 level.

*Correlation is significant at the 0.05 level.

The results of the correlation analysis revealed a moderate negative correlation between PDWM and both GAWoM and GAWM, while a strong positive correlation was observed between GAWM and GAWoM. The negative correlation between PDWM and both GAWM and GAWoM is due to the fact that, while the planned-measured dose difference is evaluated for PDWM, gamma analyses assess the agreement between the planned and measured doses. Additionally, a strong negative correlation was found between GAWM and beam-on time, and a moderate negative correlation was observed between GAWM and the modulation factor. These negative correlations can be attributed to the inverse nature of the reference metric, as higher GAWM values indicate better agreement, whereas increased beam-on time and MF may be associated with greater plan complexity. The correlations between GAWoM and both beam-on time and the modulation factor were similar to those found for GAWM. No statistically significant correlation was found between the pitch factor, gantry period, TTDF, max-LOT, mean-LOT, and the measurements, regardless of the inclusion of respiratory motion. Additionally, no significant relationship was observed between beam-on time and the fractional dose. Furthermore, a strong negative correlation was observed between PTV volume and fraction dose (−0.718, p = 0.000). As expected, this relationship indicates that the prescribed fraction dose decreases as PTV volume increases.

Since the CIRS platform is limited to one-dimensional motion, the lateral movement applied to the phantom is directly proportional to its superior-inferior movement. Consequently, the correlation analysis yielded nearly identical results for both movement directions concerning GAWM and PDWM.

## Discussion

In lung SBRT applications using the Radixact Synchrony system, two techniques can be employed to compensate for respiratory-induced target motion: FWR and LWR. The reason for selecting the LWR technique in our clinic is that it eliminates the need for invasive fiducial implantation procedures, thereby avoiding associated risks such as pain, bleeding, infection, and pneumothorax [28]. Additionally, it has been reported in the literature that when the LWR technique is used, both motion detection and dose delivery accuracy are higher compared to the FWR technique [26]. Furthermore, the Radixact Synchrony LWR technique has been shown to exhibit a lower mean target tracking error compared to other markerless lung target tracking technologies [29].

This study investigated the influence of patient-specific respiratory patterns and treatment planning parameters on the dose delivery accuracy of the Radixact Synchrony LWR tumor tracking technique for lung SBRT applications. The primary finding indicated that these variables did not have a significant impact on the dosimetric precision of this technique.

### Patient-specific QA and gamma analysis

The CCCS algorithm used for dose calculation in the Tomotherapy planning system has been shown to overestimate PTV coverage by up to 4 % for small lung tumors fully surrounded by lung tissue when compared to Monte Carlo simulations [30]. Similarly, the dose calculation accuracy of the CCCS algorithm has been reported to result in deviations of 4–5 % for small lung tumors of the island type when compared to planar dosimetric measurements using Gafchromic films and ARCCheck [31]. In our study, no significant correlation was found between the PTV volumes and dosimetric measurement results. This may be attributed to the fact that not all small tumors in our study were of the island type, with some being localized in proximity to the chest wall or mediastinum.

The PTW Detector 1500 consists of 1405 ionization chambers; however, when the merging feature is applied, the number of measurement points can increase to 2809. Both GAWoM and GAWM measurements were conducted using the same detector, with a sampling step width of 5 mm achieved by merging two measurements shifted by 5 mm, thereby fulfilling the Nyquist-Shannon sampling theorem for intensity-modulated dose distributions [20]. In our study, gamma analysis was performed using a DTA criterion of 2 mm and a DD criterion of 3 %, while Bruschi et al. demonstrated that applying the merging feature to the PTW Detector 1500 yields gamma analysis results highly comparable to those of the PTW Detector 1000 SRS, even when using the 1 mm/1 % criterion [32]. Initially, a standard patient-specific plan verification method was employed to identify potential errors arising from excessive modulation caused by forced conditions during the optimization phase, independent of the complex structure of the Synchrony tumor tracking system. As expected, a moderate negative correlation was found between GAWoM and the MF used during optimization.

Subsequently, the PDWM and GAWM analysis methods were employed to determine the impact of respiratory motion and the Synchrony system on the accuracy of planned dose delivery. The PDWM results demonstrated good agreement between the planned and measured doses. Goddard et al. also demonstrated that when motion compensation is applied using the LWR tracking method, the measured point dose deviates by only 0.3 % compared to the value measured without motion [25]. The maximum difference between planned and measured, consistent with findings in the literature, was below 2 % [26]. A strong positive correlation was observed between the PDWM and the MF. This result indicates that as the MF value set during optimization increases, the difference between the planned and measured absorbed point doses within the target also increases. Additionally, a moderate negative correlation was observed between PDWM and both GAWoM and GAWM. This result suggests that as the difference between the planned and measured absorbed point doses increases, the agreement between the planned and measured dose distributions in the gamma analysis decreases. The statistically stronger correlation between PDWM and GAWM, compared to GAWoM, can be attributed to the similarity in irradiation conditions. No significant correlation was found between the PDWM and other variables.

Upon evaluating the GAWM results, it was observed that, similar to the GAWoM findings, they were slightly lower than those reported in the literature but remained within clinically acceptable limits [25,26,33,34]. Ferris et al. assessed the dosimetric accuracy of the Synchrony FWR technique using a Delta4 Phantom mounted on a HexaMotion stage for five lung, four liver, and four pancreas patients. Consistent with our study, they reported that all measured doses achieved a gamma pass rate exceeding 90 % (3 %/2 mm/10 %TH) when compared to the planned dose [35].

An increased MF can lead to a more conformal dose distribution while enhancing the sparing of critical structures. Moreover, a higher MF provides greater flexibility in optimizing the LOT for beamlets, thereby improving dose modulation [[36], [37], [38]]. Binny et al. evaluated the effects of MF and pitch on patient-specific quality assurance using GAWoM and point dose measurements. They reported that increased MF values led to greater leaf timing inaccuracies [37]. Similarly, in our study, a moderate negative correlation was observed between MF and GAWM, consistent with the correlation observed with GAWoM. The similarity in the correlation values between MF and both GAWM and GAWoM suggests that the decrease in gamma pass rates is independent of the presence of motion [35]. Factors such as max-LOT, mean-LOT, gantry period, and pitch also contribute to plan complexity. In our study, no significant relationship was found between GAWoM and max-LOT, mean-LOT, gantry period, or pitch. Additionally, no significant correlation was identified between TTDF and GAWoM. Fusella et al. conducted GAWoM for treatment plans with higher average TTDF values (1.41 ± 0.69), applying the same gamma passing criteria as used in our study. Consistent with our findings, they concluded that TTDF had no statistically significant effect on the global gamma passing rate. Additionally, similar to our study, they demonstrated that the parameters max-LOT, mean-LOT, gantry period, and pitch did not have a statistically significant impact on gamma passing rates. However, in contrast to our results, they reported that the MF also had no significant effect on gamma passing rates [39].

A strong negative correlation was observed between beam-on time and both GAWM and GAWoM; however, no significant correlation was found between beam-on time and PDWM. This could be explained by the fact that while gamma analysis evaluates the entire treatment area, point dose measurements do not assess regions with rapid dose gradient changes. Consistent with the findings of our study, Chang et al. also reported a strong correlation for treatment time and a weaker correlation for the MF when ranking treatment planning parameters influencing patient-specific gamma analysis based on their relative importance scores. These findings, suggest that increased irradiation time may contribute to the accumulation of small errors during dose delivery [36]. Consequently, this highlights the importance of optimizing beam-on time to maintain dose delivery accuracy.

### Effect of respiratory motion on accuracy

Yang et al. evaluated the target tracking accuracy of the Synchrony system for both CyberKnife and Radixact. They reported that in both systems, tracking accuracy depends on target motion amplitude and respiratory frequency but is independent of LED motion. However, in the cases included in our study, no significant correlation was found between the dose delivery accuracy of the Radixact Synchrony system and either target motion amplitude or respiratory frequency [40]. The GAWM results were, on average, 1.3 % lower than the GAWoM results. One possible reason for the difference between the two results could be the use of different phantoms setup during the measurements. In GAWoM measurements, the PTW Octavius phantom was used, whereas in GAWM measurements, the Cheese phantom was utilized to position a high-density material near the center, enabling the Synchrony LWR technique to track it as the target tracking volume (TTV). A second possible reason for this decrease could be the minor output variation caused by the MLC shifting method used to compensate for the lateral movement of the target structure [26,41]. When the lateral movement of the target exceeds 3 mm, the Synchrony system compensates for this motion by adjusting the MLC position, resulting in minor changes in the output. No statistically significant correlation was found between the amount of lateral movement and dose delivery accuracy. For plans with lateral motion less than 3 mm, the average difference between GAWM and GAWoM was 0.56 % (range: 0.1–1.7), whereas for plans with lateral motion greater than 3 mm, this difference was 1.61 % (range: 0.2–6.4). However, in our study, the sample size with lateral movement of less than 3 mm is limited to five, providing only limited information. Additionally, to the best of our knowledge, no study has evaluated the accuracy of the Synchrony LWR technique for lateral motions smaller than 3 mm. Since the target's vertical motion was also compensated by the MLCs, if a motion platform capable of moving the target structure in the vertical direction had been used, this effect might have been more pronounced. However, in a study evaluating the Synchrony system using a platform capable of providing 3D motion, it was demonstrated that even when movements with varying amplitudes from 3 mm to 15 mm were applied across different axes, the GAWM results remained above acceptable clinical thresholds. The lowest result was 98.0 % for strict gamma evaluation criteria of 2 %/2 mm with a 10 %TH. [25].

Although the GAWM results were slightly lower than the GAWoM results, correlation analysis revealed a strong positive relationship between these two variables. Additionally, no significant relationship was identified between GAWM and respiratory pattern-related variables, such as superior-inferior and lateral motion amplitude or respiratory rate per minute.

The correlation analysis results for beam-on time in both GAWoM and GAWM were similar, indicating that the increase in beam-on time does not affect the dose delivery accuracy of the Synchrony LWR technique. Similar to the results observed for GAWoM, no significant correlation was found between planning parameters such as TTDF, max-LOT, mean-LOT, gantry period, and pitch and either GAWM or PDWM. These findings suggest that the dose delivery accuracy of the Synchrony LWR technique is not significantly affected by the complexity of the treatment plans included in this study.

The results of the correlation analyses indicate that the dose delivery accuracy of the Synchrony LWR technique is comparable to that of static target irradiation, regardless of the patient's respiratory pattern or the planning parameters used.

### Limitations of the study

First, respiratory motion was simulated using a regular sinusoidal pattern, which may not reflect the irregular breathing patterns observed in some patients. As a result, the Synchrony system's ability to adapt to irregular respiratory patterns and maintain dose accuracy could not be assessed. When the patient's breathing pattern is irregular, the Synchrony system may need to generate a new respiratory model to adapt to the changing pattern, which could interrupt the treatment. However, it has been demonstrated that even with interruptions during measurements, the dose delivered by the Synchrony system maintains acceptable accuracy [42]. Second, the same TTV structure was used in all measurements, which limits the generalizability of findings to targets with different volumes or densities. Future studies should incorporate varied TTV structures and irregular respiratory patterns to better evaluate the robustness of the Synchrony system under more clinically realistic conditions.

## Conclusion

This study demonstrates that, with appropriate patient selection, respiratory patterns and treatment planning parameters in lung SBRT patients have no significant impact on the dose delivery accuracy of the Lung with Respiratory technique of the Radixact Synchrony System.

## Declaration of competing interest

The authors declare that they have no known competing financial interests or personal relationships that could have appeared to influence the work reported in this paper.
